# Treatment of Chronic Gonorrhœa by Means of Grooved Sounds

**Published:** 1886-06

**Authors:** 


### Treatment of Chronic Gonorrhœa by Means of Grooved Sounds.

This method, introduced by Casper, unites the advantages of mechanical with medical treatment. Conical sounds ten inches in length, having longitudinal grooves one-sixteenth of an inch in depth, are used. Into these grooves a medicated ointment, solid at ordinary temperatures, is rubbed. Casper has used various ointments, among others a three per cent. ointment of resorcin, but he considers Unna's ointment the best. The following is the formula:

| | | PARTS. |
|---|---|---|
| ℞ | Argenti Nitrat., - - - - | 1 |
| | Bals. Copaibæ, - - - | 2 |
| | Ol. Theobromæ, - - - - | 100 |

M. Sig. Pomade.

This ointment melts at the normal temperature of the body, and liquifies when introduced into the urethra. Flexible bougies of the ordinary variety are first used to prepare the urethra, then the medicated sounds are introduced.

Casper has, in this way, cured thirty cases of obstinate gonorrhœa. The average number of treatments required was ten, the highest number twenty.—*Berlin Klinische Wochenschrift.*

